# Minute hands of clocks indicating the same time are not perceived as haptically parallel

**DOI:** 10.1038/s41598-018-21415-9

**Published:** 2018-02-14

**Authors:** Astrid M. L. Kappers

**Affiliations:** Department of Human Movement Sciences, VU Amsterdam, Amsterdam, 1081 BT The Netherlands

## Abstract

Many studies have already shown that a large idiosyncratic orientation difference is needed to perceive two bars that are far apart as haptically parallel. There exist also strong indications that if such bars are imagined to be minute hands of clocks, errors made in clock time estimates and clock time settings are much smaller. The current study investigated this seemingly discrepancy. Participants partook in three experiments: parallel setting, clock time estimate and clock time setting, in this order. As the individual parallel settings were used in the subsequent clock time estimate experiment, and the estimated clock times in the clock time setting experiment, the deviations could be compared directly. In all three experiments, the deviations were systematic and idiosyncratic, and consistent with a biasing influence of an egocentric reference frame. However, the deviations in the two clock time experiments were indeed much smaller than in the parallel setting experiment. Task instruction and strengthened focus on an allocentric reference frame are the most likely explanations. These findings provide fundamental insights in the processing of spatial information. Taking these findings into account when designing haptic devices may make these more intuitive.

## Introduction

Many studies have already shown that human perception of spatial relations is not veridical e.g.^1–6^. In these studies, the task of the blindfolded participants is to change the orientation of a test bar in such a way that the bar feels parallel to a similar bar at a different location. Depending on the exact experimental conditions, the difference in orientation can be as large as or even larger than 90°. Interestingly, the *size* of these deviations strongly depends on the participant, but the *direction* of the deviation is consistent over all participants: a test bar placed to the right of a reference bar has to be rotated clockwise in order to feel as parallel. In a study^2^ in which 68 participants performed 8 trials each, the average deviation was 41°.

Substantial evidence is presented that the deviations in the parallel setting experiment are caused by the biasing influence of an egocentric reference frame^4,5,7^. There exist many different egocentric reference frames, such as the body, the hand, the head, the eye, etc. e.g.^8–10^. In this type of experiments, the hand seems to contribute most to this egocentric reference frame, but also arm and body have some influence e.g.^4,11^.

Experimental tasks that seem closely related to the parallel setting task are the clock time estimate^12–15^ and clock time setting^13,14^ tasks. In the clock time estimate task, blindfolded participants have to judge the clock time in minutes of a bar presented to them; in the clock time setting task they have to adjust the bar to an instructed number of minutes^14^ or degrees^13^. These tasks can be performed using the same set-ups as the parallel setting task. In these experiments, the location of the virtual clocks is systematically varied, but their orientation is always parallel in an allocentric reference frame (that is, hour hands pointing to 12 o’clock are all parallel to the midsagittal plane).

Zuidhoek *et al*.^12^ investigated the clock time estimate task on a horizontal plane. Four test locations were used, two to the right and two to the left of the midsagittal plane. Both right and left hands were used to estimate the clock times at all four locations. They found that if the reaching hand had to be rotated more with respect to the allocentric reference frame, the errors became larger. They also found that the deviations seemed smaller than in the typical parallel setting experiment, even after doubling the deviations to account for the fact that in the clock time estimate experiment only one hand is involved in a trial. However, since the deviations in the parallel setting experiment strongly depend on participant and because the clock locations were different from the locations used in the parallel setting experiment, a direct comparison between the sizes of the deviations in these two experimental tasks could not be made.

Hermens *et al*.^13^ investigated all three experimental tasks, parallel setting, clock time estimate and clock time setting, on a frontoparallel plane. They concluded that the deviations in the two clock time experiments were substantially smaller than in the parallel setting experiment and that the direction of the deviations was not consistent over participants. It should be noted, however, that in two of the experiments they used only four different orientations, namely two cardinal directions (0° and 90°) and two special oblique orientations (45° and 135°). Although the same orientations have been used many times in parallel setting experiments, there is also evidence that these orientations are ‘special’ and therefore possibly not representative for overall performance e.g.^16,17^. Hermens *et al*.^13^ also concluded that the deviations in the parallel setting task could not be explained by a combination of an error in the perception of the reference bar and an error in the production of the orientation of the test bar. Unfortunately, mostly different participants partook in the various experiments. As the sizes of the errors depend so much on participant, this conclusion should be taken with some caution.

Also Zuidhoek *et al*.^14^ investigated all three experimental tasks. Although the participants in all three experiments were the same, many other experimental details, such as stimulus orientations and locations, were different. Moreover, the focus was more on sex and hemispheric differences, than on differences and similarities between the three tasks. They report systematic deviations in all three tasks, the deviations in the parallel setting task being largest. However, because of the mentioned differences, the exact relationship between the deviations in the three tasks was left unexplored. Postma *et al*.^15^ extended this research by also testing early blind and late blind participants in the parallel setting and clock time estimate tasks. They found that the blind participants made significantly larger errors in the clock time estimate task, signifying the importance of vision for such a task, even if vision cannot be used for the task itself.

The aim of the current study is to investigate the similarities and differences in the errors made in the three different experiments in a more systematic way than in the previous studies. The major research question is whether the deviations in the two clock time experiments are indeed smaller, taking into account that clock time experiments are done unimanually whereas the parallel setting experiment is done bimanually. This is not a trivial question, as the somewhat counterintuitive logical consequence of smaller deviations is that the minute hands of two clocks that indicate the same time are not perceived as parallel. If the errors are indeed smaller in the clock time experiments, the differences need to be characterized further. Are the differences only quantitative in nature, or is there also a qualitative difference? And how, if at all, are the errors in the clock time estimate and clock time setting experiments related?

Participants first have to make a series of pairs of bars parallel. Subsequently, the bar orientations that they set as parallel are presented to them in a clock time estimate experiment (but in random order). If the reference frames in which the two tasks are performed are the same, the estimated clock times should be the same for pairs of bars that were set as parallel. In a third experiment, the participants are instructed to set bars in the orientations estimated in the second experiment. As in all three experiments the same persons participated, direct comparisons between the errors made in the experiments are possible. The results will shed light on the use of possibly different reference frames in the three experiments.

## Results

### Experiment 1: Parallel setting

**Figure 1:**
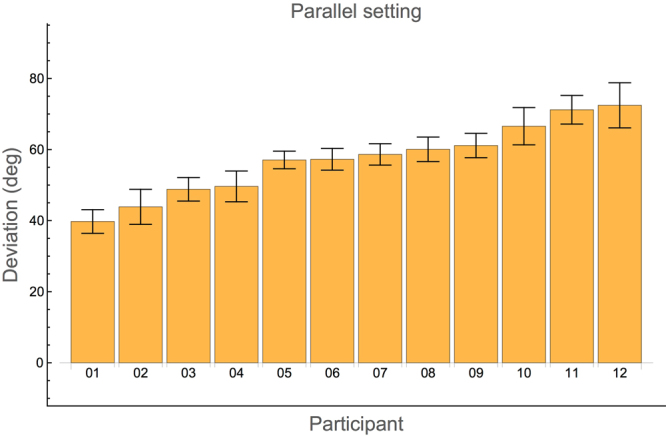
Average signed deviations in the parallel setting experiment for each participant. The deviation is defined as the orientation of the left bar minus the orientation of the right bar, irrespective of whether the left bar was the reference or the test bar. Positive deviations indicate that the right bar is rotated clockwise with respect to the left bar in order to feel the two bars as having parallel orientations. Error bars indicate standard errors. The results of the participants are ordered according to their mean deviation. The same order is used in all other figures.

As in most of the previous studies, the deviations in this experiment are determined as the orientation of the left bar minus the orientation of the right bar e.g.^2^. In Fig. 1, the average signed deviation of each participant is shown. The results of the participants are sorted according to their average deviation. This order is kept the same in all subsequent figures. Consistent with previous studies, all deviations are positive, meaning that the right bar has to be rotated clockwise with respect to the left bar in order to be felt as parallel. Thus, if the right bar was the reference bar, the left bar was rotated anticlockwise. Averaged over all participants, the deviation is 57 ± 10°. The deviations did not depend on the reference hand: 57 ± 10° and 58 ± 11° for left and right hand used as reference, respectively. To illustrate how large such a deviation actually is, Fig. 6 in the Methods section shows a typical setting.

### Experiment 2: Clock time estimate

**Figure 2:**
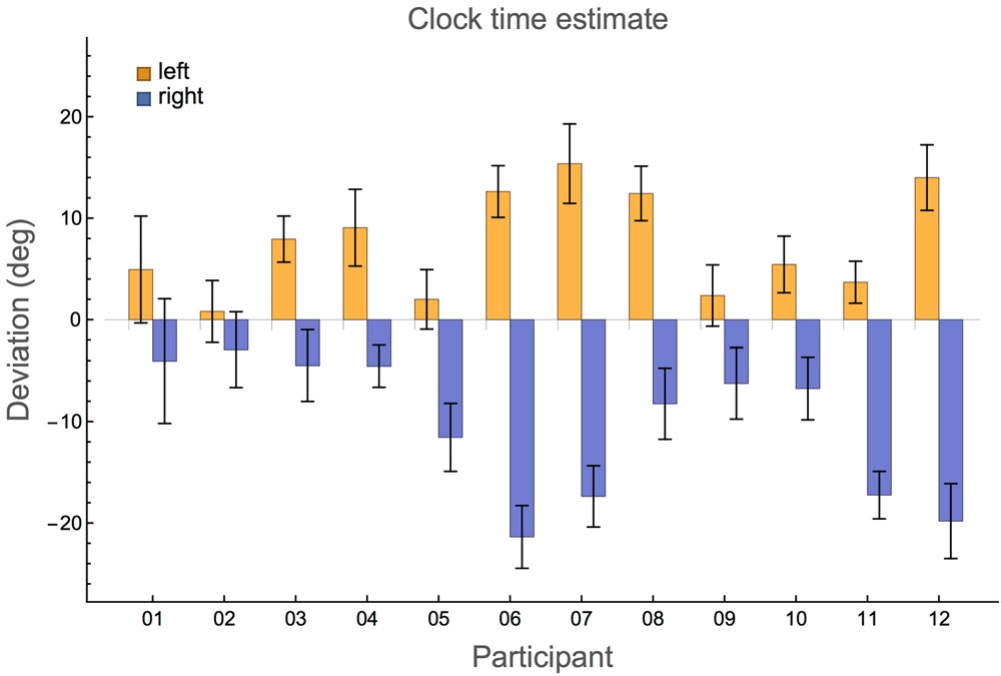
Average signed deviations in the clock time estimate experiment for each participant and both hands. The deviation is defined as the reference orientation minus the estimated orientation (both in degrees). Error bars indicate standard errors. The results of the participants are shown in the same order as in Fig. 1, but note the difference in scale.

In Fig. 2 the deviations in the clock time estimate experiment are shown for each participant. The deviations of the left hand estimates are all positive (mean 7.6 ± 5.1°), those of the right hand all negative (mean −10 ± 6.8°). A positive deviation on the left side means that the participant responded with a number of minutes that is *larger* than the reference time. Likewise, a negative value on the right sight means that the participant answered with a *smaller* number of minutes than the reference. One-sample two-tailed *t*-tests showed that both these values were significantly different from 0 (*t*(11) = 5.2, *p* < 0.0004 and *t*(11) = 5.3, *p* < 0.0003 for left and right hands, respectively). The sizes of the left and right hand deviations did not differ significantly (paired *t*-test, *t*(11) =−1.7, *p* = 0.12, right hand deviations multiplied by −1). Note that 6° is equal to 1 minute, thus even though the deviations are significant, they are quite small.

### Experiment 3: Clock time setting

**Figure 3:**
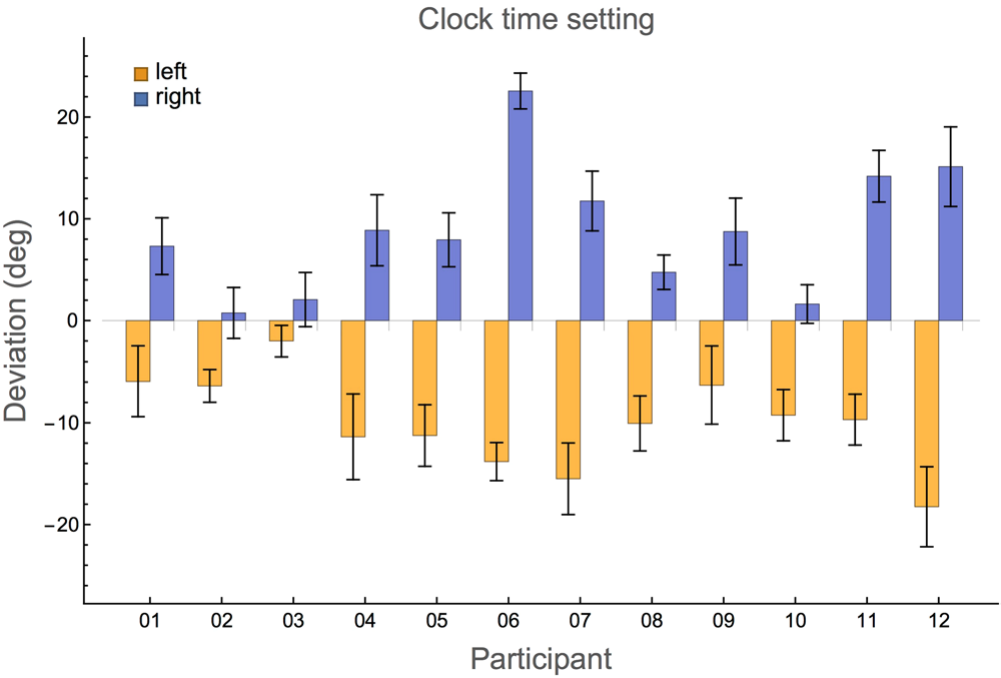
Average signed deviations in the clock time setting experiment for each participant and both hands. The deviation is defined as the instructed reference orientation minus the orientation set by the participant (both in degrees). Error bars indicate standard errors. The results of the participants are again shown in the same order as in Fig. 1, but again note the difference in scale.

Figure 3 shows the deviations in the clock time setting experiment for each participant. With the definitions used, all deviations of the left hand are negative (mean over all participants −10 ± 4.5°); for the right hand these are all positive (8.8 ± 6.4°). A negative deviation at the left side means that the participants set the bar at an *earlier* time in minutes than the instructed reference time. Likewise, a positive deviation at the right sight shows that participants set the bar at a *later* time than instructed. One-sample two-tailed *t*-tests showed that both these values were significantly different from 0 (*t*(11) = 7.7, *p* < 0.00001 and *t*(11) = 4.8, *p* < 0.0006 for left and right hands, respectively). The sizes of the left and right hand deviations did not differ significantly (paired *t*-test, *t*(11) = 0.86, *p* = 0.41, right hand deviations multiplied by −1).

### Comparison between experiments

**Figure 4:**
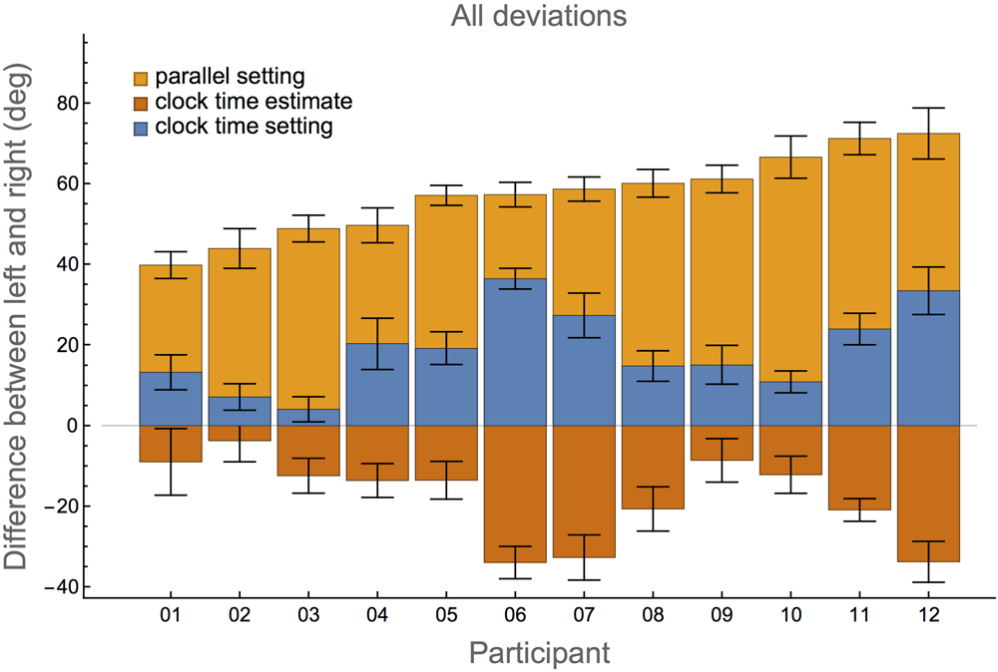
Difference between left and right hand settings and estimates for all three experiments. Note that all bars are drawn from zero, thus it is immediately clear that the sizes of the deviations in the parallel setting experiment are much larger than those in the other two experiments. Error bars indicate standard errors.

In the parallel setting experiment, always both hands are involved in determining the deviations, whereas in the other two experiments, the deviations were determined for each hand separately. However, as one of the main questions in this study is to investigate whether or not the deviations in the clock time estimate and clock time setting experiments are indeed smaller than in the parallel setting experiment, somehow a fair comparison has to be made. Due to the design of the experiments, each reference in the clock time estimate and clock time setting experiments can be matched to a reference in the parallel setting experiment. Taking together the deviations of the left and right hands that belong to settings in the parallel setting experiment, two-handed deviations can be determined for all three experiments. This is explained in more detail in the Methods section. The resulting deviations are shown in Fig. 4. The data for the parallel setting experiment are identical to those presented in Fig. 1. The data for the other experiments can be inferred from Figs. 2 and 3 by taking the difference between the deviations of the right hand and those of the left hand. For example, the deviations of participant 1 in the clock time estimate experiment are 4.9° for the left hand and −4.1° for the right hand (see Fig. 2). Thus, the average total deviation is −4.1°–4.9° =−10°, which is the value that can be seen in Fig. 4.

As reported above, the average deviation in the parallel setting experiment is 57 ± 10°. The two-handed deviation in the clock time estimate experiment is −18 ± 11° and in the clock time setting experiment 19 ± 10°. Note that the signs of the deviations are a direct consequence of the definitions. One-sample two-tailed *t*-tests showed that the deviations in the parallel setting experiment are significantly larger than in the two other experiments (*t*(11) = 13.7, *p* < 0.00001 and *t*(11) = 13.3, *p* < 0.00001 for comparison with clock time estimate (deviations multiplied by −1) and clock time setting, respectively). The two-handed deviations of the clock time estimate and clock time setting experiments do not differ significantly (*t*(11) = 0.57, *p* = 0.58).

A final question is whether the deviations in the various experiments are correlated. In other words, do participants that have large deviations in the parallel setting experiment, also have large deviations in the other two experiments (and vice versa)? The Pearson correlations *r* between the two-handed deviations in the parallel setting experiment with the clock time estimate and clock time setting deviations turn out to be not significant (*r* =−0.55, *p* > 0.06 and *r* = 0.51, *p* > 0.09, respectively). The correlation between the clock time estimate and clock time setting deviations was highly significant (*r* =−0.88, *p* < 0.0002).

## Discussion

The major research question of this study was whether the deviations in the clock time experiments were indeed smaller than in the parallel setting experiment. The outcome is very convincingly yes, the deviations in both clock time experiments are much smaller. Interestingly, this means that clock hands of clocks that are judged as indicating the same time, do not feel as parallel.

In all three experiments systematic deviations were found. The *size* of these deviations was participant-dependent, but the *direction* of the deviations was very consistent over participants. Deviations in the parallel setting experiment were also consistent with many earlier studies e.g.^1–6^ and as always, they were quite substantial (on average the right bar needs a clockwise rotation of 57° with respect to the left bar in order to be felt as parallel). The deviations in the clock time estimate and clock time setting experiments were significantly smaller than in the parallel setting experiment (two-handed deviations were −18° and 19°, respectively). This is also in accordance with the previous studies, that were performed under different conditions^12–15^.

**Figure 5:**
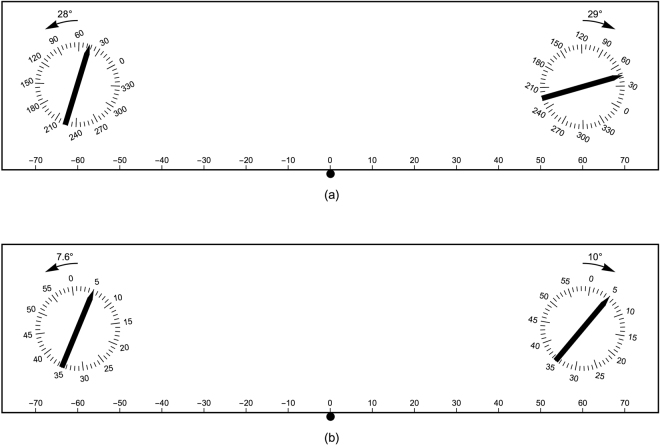
Schematic illustration of rotated reference frames. The figures show the rotations of the reference frames in the three experiments. Each hand has its own reference frame. The rotations needed are participant-dependent. (**a**) Parallel setting experiment. As the deviations for the left and right hand cannot be determined independently in this experiment, the rotation indicated is somewhat arbitrarily taken as half that belonging to that hand as reference. Averaged over all participants, the bars that are shown are perceived as haptically parallel. (**b**) Clock time estimate and clock time setting experiments. The numbers indicate the rotations needed in the clock time estimate experiment, but the rotations in the clock time setting experiment are quite similar, namely 10° and 8.8°, for left and right sides, respectively. Averaged over all participants, the bars that are shown are estimated as having the same clock time. Note that the same rotated clock frames lead to deviations of opposite sign in the clock estimate and clock setting experiments. In both figures, the curved arrows are not to scale.

The deviations in the parallel setting experiment can be interpreted as if the reference frames belonging to the two hands are rotated with respect to the world/table reference frame. This is schematically illustrated in Fig. 5a. As the deviations cannot be attributed to the hands separately (in each trial always both hands were involved), only for the illustration the rotations are somewhat arbitrarily determined as half of the deviation when the hand was used as reference (28° anticlockwise for the left hand and 29° clockwise for the right hand). The deviations in the clock time estimate experiment are also consistent with rotated reference frames. However, in this case the rotations are much smaller than in the parallel setting experiment. In this experiment the deviations were determined for each hand separately, thus these rotations can be schematically illustrated directly (see Fig. 5b). The two bars shown indicate which orientations participants on average estimated to be a clock time of 5 minutes with their left and right hands. Finally, also the deviations in the clock time setting experiment are consistent with rotated reference frames. Moreover, the rotations needed are also fully consistent with those obtained in the clock time estimate experiment (apart from small non-significant differences). Note that the difference in sign of the deviations in the clock time estimate and clock time setting experiments originates from the definition of the deviations: reference orientation - test orientation (in degrees). A clock time of 5 minutes on the left side of the table frame, will be *estimated* to be somewhat later, hence resulting in a positive deviation (in degrees); with the same rotation of reference frame, *setting* a clock time of 5 minutes will result in a somewhat earlier time in the table reference frame, resulting in a negative deviation.

Hermens *et al*.^13^ did not find any systematic deviations in the two clock time experiments, which is different from the findings in the present study. However, this is probably due to the many differences in experimental conditions, such as frontoparallel versus horizontal plane, and location of the bars with respect to the participant. Especially this latter aspect seems of relevance, as in their set-up also the deviations in the parallel setting experiment were smaller. Based on the current results, it might be expected that if parallel setting deviations are smaller due to the experimental conditions, also clock time deviations will be smaller. As these were already quite small in the current study, one might expect averaged deviations close to zero in the Hermens *et al*. study. Indeed their averaged deviations were not significantly different from 0° in the two clock time experiments. The deviations found in the present study are in line with those reported in other earlier studies of the clock time estimate experiment^12,14,15^ and the clock setting experiment^14^.

The conclusion emerging from the results is that the deviations in both the parallel setting experiment and the two clock time experiments are caused by the biasing influence of an egocentric reference frame, but not in the same manner. This difference is mainly *quantitative* in nature, as the deviations are biased in the same direction. Moreover, although the correlations between the parallel setting deviations and the clock time deviations were not significant at the 5% level, they were at the 10% level. Therefore, there is no substantial evidence that the differences are also of a *qualitative* nature.

What might have caused this quantitative difference? One important difference is that in the clock time experiments, participants more explicitly have to focus on the allocentric reference frame than in the parallel setting experiment. The virtual clocks exist in the allocentric world, not in their egocentric reference frame. The clock time tasks encourage participants to mentally imagine such clocks, and such instructions strengthen the use of an allocentric reference frame^15^, which in turn would cause the errors to reduce. Also the strategies used may differ. In the clock time experiments, some participants were observed trying to align their hand with an imaginary clock hand pointing to 12 o’clock, which is quite different from participants trying to align their hands with the bar, as often observed in the parallel setting experiment.

There are other variations of the parallel setting task that also showed reduced errors. In a parallel setting experiment, Van Mier^5^ allowed participants to look at their test hand, while the hand touching the reference bar was covered. The errors made in this condition were still systematic, but reduced to about half the size of the parallel settings. Such a reduction was also found if only a protractor without a test bar was shown and participants had to respond with a coded orientation^6^. A further reduction of the size of the deviations was found when participants with vision of their test hand had to draw a line instead of setting a test bar^5^. In all these cases, visual information is involved. Indeed, in a purely visual parallel setting experiment in which participants were allowed to see both bars but were not allowed to touch them, also much smaller, but still systematic deviations were found^18^. However, even if noninformative vision was present, that is, vision of the surroundings but not of the stimuli and hands, a significant reduction of the deviations was found e.g.^19,20^. Finally, in a clock time estimate experiment, blind participants performed worse than blindfolded sighted participants^15^, which presents additional evidence that the availability of vision plays an influencing role in such tasks.

Cuijpers *et al*.^21^ investigated the metrics of visual and haptic space based on parallel setting experiments in both modalities. By making some assumptions and ignoring differences between cardinal and oblique reference orientations, they could show that the intrinsic geometries of both visual and haptic space are Euclidean. As the differences between the two clock time experiments and the parallel setting experiment seem merely quantitative in nature, the results of all three experiments are consistent with the haptic model of Cuijpers *et al*.^21^.

Summarizing, based on the present and past studies, it is clear that task instructions that seem equivalent in an allocentric reference frame, may lead to quite different results in actual performance. Apparently, the biasing influence of egocentric reference frames may strongly vary in strength. Understanding and mapping this use of different reference frames (or the different weighing between allocentric and egocentric reference frames) in different tasks is not only of fundamental interest, it has also an increasingly important practical utility. Several studies showed that making the interaction with haptic devices more intuitive will improve human performance e.g.^22–24^. In many teleoperating tasks, spatial relations of objects in the environment play a significant role. As shown again in the current study, the perception of spatial relations is susceptible to idiosyncratic distortions that strongly depend on task instructions. Understanding what is intuitive and what is not, and in which circumstances and under which task instructions, will help improving interaction performance and as a consequence, also the safety of both human and machine.

## Methods

### Participants

Twelve PhD students and postdocs from other groups of the Department of Human Movement Sciences (6 females), who were unaware of the research questions and unfamiliar with the experimental set-up, volunteered to take part in the experiments. All but one were right-handed as assessed by means of a standard questionnaire^25^. All signed an informed consent form. All participants performed the three experimental tasks. The research program was approved by the Ethical Committee of the Department of Human Movement Sciences, Faculty of Behavioural and Movement Sciences, VU Amsterdam. The experiments were carried out in accordance with the approved guidelines.

### Set-up and stimuli

**Figure 6:**
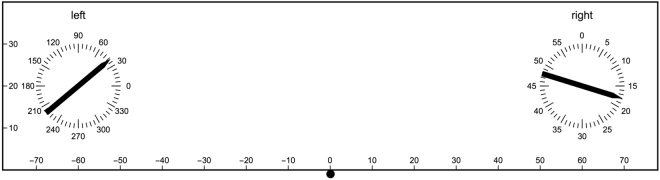
Schematic top view of the set-up. The small solid circle indicates the center position of the participant. On the left, the division in degrees is shown, on the right in minutes. Note that the actual protractors had ticks for each degree, whereas minutes were not indicated. Only in the parallel setting experiment, the two bars were present. The illustrated difference in the orientations of the bars shows the average deviation obtained in the parallel setting experiment in this study; this is what participants, on average, perceived as parallel bars.

The set-up was used and described in many earlier studies e.g.^1,26^. It consisted of a table of height 78 cm on which an iron plate and printed protractors were fixed. The two protractors used in the current experiment were positioned at locations (−60, 20) and (60, 20), where position (0, 0) was right in front of the participant (all units are given in cm, see Fig. 6). Aluminum bars of length 20 cm with one pointed end could be placed on the protractors. The bars could pivot on a small pin in the center of the protractor. Small magnets fixed to the bars increased friction (thanks to the iron plate).

### Procedure

When the participants entered the experimental room, the set-up was covered with a cloth. They sat down on a stool at the correct position. The stool could be adjusted in height so that the shoulders of the participant were approximately at 110 cm. They could feel a small screw at the side of the table where they should center themselves. Before the start of the experiments, it was first tested whether the participants were familiar with the notion ‘parallel’. A pen was placed on the covered table in a random oblique orientation. They were asked to position a second pen in such a way that it was parallel to the first pen with the tips of the pens pointing in the same direction. Next, they had to do the same task but with the pens far apart. All participants passed this test.

Subsequently, they were asked to blindfold themselves. The cloth was removed from the table and they were informed that they would start with the first experiment, making two bars parallel. At this point, they knew that they had to participate in three haptic experiments, but they did not yet receive any information about the other two experiments.

The maximum time for a trial was 10 s in all experiments. A well audible click signified the end of a trial, and at that moment participants were no longer allowed to touch the bars. In the majority of the trials, this time limit was (more than) sufficient. The total time involved was about 45 minutes for each participant. During the experiments, they never received any feedback about their performance.

The experiments were always performed in the same order: (1) parallel setting, (2) clock time estimate, and (3) clock time setting, but the order of trials within an experiment was random. After finishing all three experiments, participants were asked to do one more parallel setting, after which they were allowed to remove their blindfold, so that they could see their own setting. As all participants have rather large deviations in this task, the reaction to their own setting provided an extra test whether they correctly understood ‘parallel’. All participants also passed this test.

In all three experiments, participants were free in their choice of hand movement strategy. Thus, they did not receive any instructions on whether to use their whole hand or just their fingers, whether or not to align their hands with the bars, etc. They were, however, explicitly instructed to take note of the pointed ends of the bars (like in the practice trial with the pens). In practice, participants used their fingers to check on which side the pointed ends of the bars were and subsequently, they used their whole hands to orient the bars.

### Experiment 1: Parallel setting

In this experiment, participants were asked to rotate a test bar in such a way that it felt parallel to the reference bar. The orientations that were used for the reference bar were 5, 10, 20, 25, 35, 40, 50, 55 minutes, which corresponds to 60°, 30°, 330°, 300°, 240°, 210°, 150°, and 120°, respectively on the protractors (see Fig. 6). This selection of reference orientations avoids the cardinal orientations 0°, 90°, 180°, and 270°. Note that this study does not focus on nor is interested in the effect of reference orientation. The test bar had a random orientation, which was never the orientation set in the previous trial. The reference bar could be positioned on both the right and left sides. When the reference position was on the right, the test bar was positioned on the left and vice versa. All 16 combinations of reference position and orientation were presented once in random order to the participant. The participant had to touch both bars at the same time (right hand on the right side, left hand on the left side) and was only allowed to adjust the orientation of the test bar. At the start of the trial, the experimenter clearly stated which bar was the reference bar.

At the end of each trial, the experimenter entered the test orientation in a computer program, and subsequently oriented the reference bar at the correct position for the next trial. The experiment started with one or two practice trials without feedback to familiarize the participant with the task.

### Experiment 2: Clock time estimate

In this experiment, the participants were told that they had to imagine clocks printed on the table at the position of the bars. They were told that the clocks were aligned and that the hour-clock-hand would point straightforward if the time were twelve o’clock. Their task was to estimate the orientation of the test bar in minutes. Although not asked explicitly, all participants answered in whole minutes.

The reference orientations used in this experiment corresponded with the orientations given and obtained in the parallel setting experiment. Unknown to the participant, all reference orientations used in the parallel setting experiment and all the test settings of the participant were used here at the corresponding position. In this way, it could be analysed directly whether bars that feel parallel are also estimated to have the same clock time. Because of this design, only the 16 orientations originating from the reference bar orientations in the parallel setting experiment were the same for all participants. All 32 trials were presented in random order. The participant used only one hand at a time. The experiment started with one practice trial.

### Experiment 3: Clock time setting

In this third experiment, participants were asked to orient the test bar so that it corresponded with the number of minutes mentioned by the experimenter. The minutes used as reference were the minutes mentioned by the participant in the Clock time estimate experiment (but again, this was unknown to the participant). As a consequence of this design, the instructed clock times in this experiment were different for each participant. Also this experiment consisted of 32 trials, 16 presented on the right side and 16 on the left side. No practice trials were needed.

### Data analysis

The data analyses will be done in degrees, as that is the unit that could be read off from the protractors and for convenient comparison with previous studies. So even if the task of the participant had to do with clock times, i.e. minutes, computations will be done in degrees.

The data of the parallel setting experiment will be analysed in the same manner as in most previous studies. As in a trial always both hands are involved, deviations cannot be assigned to separate hands. Therefore, the deviations are defined as the orientation of the left bar minus the orientation of the right bar. Positive deviations mean that the right bar is rotated clockwise with respect to the left bar, or, if the right bar was the reference bar, that the left bar is rotated anticlockwise with respect to the right bar. Previous studies always showed large positive deviations e.g.^2^. The possible influence of reference orientation is not topic of study here and will therefore not be analyzed.

In the clock time estimate experiment, deviations can be analyzed for the two hands separately. Here, the deviations are determined as the reference orientation minus the estimated orientation. As the computations are done in degrees as shown in Fig. 6, a positive deviation means that the number of minutes estimated by the participant is larger (clockwise rotation) than the reference orientation in minutes.

In order to compare the size of the deviations in the clock time estimate experiment with those obtained in the parallel setting experiment, it is also relevant to determine a combined deviation for the left and right hands. For this purpose, left and right estimates belonging to reference orientations that were obtained in the parallel setting experiment as parallel will be taken together. If performance in the two experiments were fully consistent, the estimated clock times of the left and right bars that are perceived as being parallel, will be similar, even if the bars have different orientations. As a consequence, the clock time estimate deviation would also be similar to that in the parallel setting experiment. On the other hand, if clock time estimates are much closer to veridical, as might be expected on the basis of previous experiments^12,13^, the clock time estimate deviation will be smaller. The clock time estimate deviation is determined as the left clock time estimate minus the right hand estimate minus the difference in reference orientation (due to the parallel setting deviation belonging to these trials). This is the same as the right hand deviation minus the left hand deviation. In this way, a positive value indicates that the right hand deviation is relatively more clockwise than the left hand deviation.

In the clock time setting experiment, the deviations are also analyzed for each hand separately. Here, the deviation is defined as the instructed (reference) orientation minus the set orientation (again in degrees). A positive deviation means that the orientation of the bar is set more clockwise than the instructed reference orientation. A combined left hand, right hand deviation can be determined for comparison with the other two experiments. The clock time settings belonging to the clock time estimates that belonged to bars that were perceived as parallel are taken together. The clock time setting deviation is determined as the left clock time setting minus the right clock time setting minus the difference in reference orientations, which is the same as the right hand deviation minus the left hand deviation. In this way, a positive value indicates that the right hand deviation is relatively more clockwise than the left hand deviation.

Averages mentioned in the text are given as mean ± standard deviation, unless explicitly stated otherwise. In the statistical tests, a significance level of 5% is used throughout. Data used in statistical tests always fulfilled the requirement of normality.

### Data availability

The datasets generated during and/or analysed during the current study are available from the corresponding author on reasonable request.
